# A retrospective cross-sectional study: fresh cycle endometrial thickness is a sensitive predictor of inadequate endometrial thickness in frozen embryo transfer cycles

**DOI:** 10.1186/1477-7827-11-35

**Published:** 2013-05-10

**Authors:** Patricia T Jimenez, Samantha B Schon, Randall R Odem, Valerie S Ratts, Emily S Jungheim

**Affiliations:** 1 Department of Obstetrics and Gynecology, University of Texas Southwestern Medical Center, Dallas, TX, USA; 2 Department of Obstetrics and Gynecology, Washington University School of Medicine, Washington, DC, USA

**Keywords:** Endometrial thickness, Frozen embryo transfer, Estrogen supplementation

## Abstract

**Background:**

The purpose of this study is to assess predictors of inadequate endometrial cavity thickness (ECT), defined as < 8 mm, in frozen embryo transfer (FET) cycles.

**Methods:**

This is a retrospective cross-sectional study at an academic fertility center including 274 women who underwent their first endometrial preparation with estradiol for autologous FET in our center from 2001-2009. Multivariable logistic regression was performed to determine predictors of inadequate endometrial development in FET cycles.

**Results:**

Neither age nor duration of estrogen supplementation were associated with FET endometrial thickness. Lower body mass index, nulliparity, previous operative hysteroscopy and thinner fresh cycle endometrial lining were associated with inadequate endometrial thickness in FET cycles. A maximum thickness of 11.5 mm in a fresh cycle was 80% sensitive and 70% specific for inadequate frozen cycle thickness.

**Conclusions:**

Previous fresh cycle endometrial cavity thickness is associated with subsequent FET cycle endometrial cavity thickness. Women with a fresh cycle thickness of 11.5 mm or less may require additional intervention to achieve adequate endometrial thickness in preparation for a frozen cycle.

## Background

As in vitro fertilization (IVF) culture techniques continue to improve, the number of embryos transferred in each cycle decreases. Transferring fewer embryos is a key component in preventing multiple gestations and their associated complications. As a result, fresh IVF cycles often lead to supernumerary embryos. Cryopreservation of embryos created during fresh IVF cycles provides a less expensive and time-intensive opportunity for pregnancy. In addition, vitrification has proven to be a superior method for cryopreservation, further improving frozen embryo transfer (FET) pregnancy rates [1,2]. Therefore, optimization of the FET cycle is crucial.

For FET, the endometrium is frequently artificially prepared with estrogen and progesterone supplementation in order the match the endometrial stage during the critical implantation window [3]. There are a number of protocols used in endometrial preparation [4]. The optimal endometrial thickness is unclear. However, several studies suggest a thickness < 8 mm is associated with implantation failure in both fresh and frozen embryo transfer cycles [5-8]. Given the resources involved in IVF and FET cycles, optimization of an individual’s treatment plan is critical. Women preparing for FET will often require additional estrogen supplementation, or other intervention, if their endometrium is thin. The objective of this study is to determine predictors that may identify women who require additional estrogen supplementation to attain adequate endometrial thickness in preparation for FET cycles to inform future FET cycle planning.

## Methods

This was a retrospective cross-sectional study. STROBE guidelines for cross-sectional studies were followed [9]. Patients who underwent fresh autologous oocyte retrieval with embryo transfer cycle and a subsequent medicated FET cycle within our center between January 2001 and February 2009 were eligible. Only the first FET cycle in which oral estradiol was used for endometrial preparation was included. Embryos were cryopreserved using a slow freeze protocol. All frozen embryo transfers were performed at the blastocyst stage. Donor oocyte recipients and gestational carriers were excluded. The Institutional Review Board at Washington University School of Medicine approved this study.

Per protocol in our center for blastocyst stage transfers, patients begin oral estradiol 2 mg three times a day on cycle day 1. After at least 12 days of oral estradiol, a serum progesterone and ultrasound is performed to measure endometrial cavity thickness (ECT). If progesterone is < 2.5 ng/ml and endometrial thickness is at least 8 mm, patients begin IM progesterone two days later and embryo transfer is performed after five days of progesterone. If endometrial thickness is < 8 mm, estradiol replacement is continued based on physician preference as oral estradiol 2 mg three times a day or the addition of transdermal estrogen (0.1 mg patches) with re-evaluation after one week.

Medical charts of subjects were reviewed and the following data were collected: age, ethnicity, infertility diagnosis, gravity, parity, body mass index (BMI), dosage and duration of estradiol supplementation, prior uterine surgery, endometrial thickness in the fresh and FET cycle, number of embryos transferred, and pregnancy outcome of the fresh and FET cycle.

Three hundred and six subjects met inclusion criteria. There was incomplete data available on 15 patients and 17 patients had cryopreserved embryos from two fresh cycles transferred during the FET cycle. The remaining 274 subjects were included in the analysis. Standard univariate analyses were performed to determine associations between inadequate endometrial development (defined as < 8 mm) and the following variables: age, duration of estrogen supplementation, BMI, history of prior pregnancy, history of prior operative hysteroscopy and endometrial thickness in fresh embryo transfer cycle. Variables with a p-value <0.05 in the univariate analyses were entered into a logistic regression model assessing their association with inadequate endometrial development. All analyses were performed in the Statistical Package for the Social Science (SPSS) version 16.0 (SPSS Inc., Chicago, IL).

## Results

**Figure 1:**
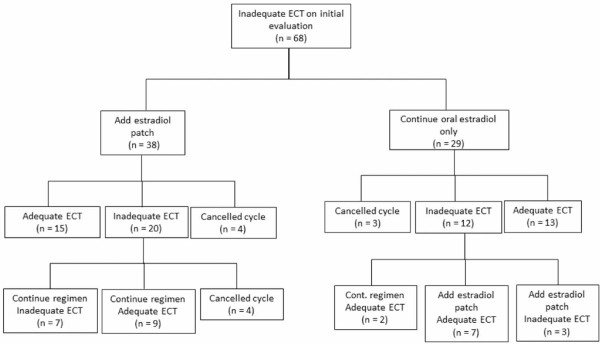
Flow chart of the management of patients with inadequate endometrial cavity thickness (ECT) on first evaluation.

The mean ECT for all FET cycles was 9.2 ± 2.7 mm. Neither age nor duration of estrogen supplementation was associated with adequate or inadequate endometrial development (Table 1). Subjects with inadequate endometrial development during FET had a lower BMI (22.7±3.51 vs. 25.62±5.54 kg/m^2^; p <0.0001), they were less likely to have a prior pregnancy (70.6% vs. 87.4%; p=0.001), they were more likely to have a history of uterine surgery (7.4% vs. 1.9%; p=0.04) and a thinner endometrial lining during a fresh cycle (10.05±1.89 vs. 12.93±3.12 mm; p<0.001). The majority of patients (67%) with an inadequate endometrial thickness on initial evaluation ultimately had appropriate endometrial development after further estradiol supplementation (Figure 1).

**Table 1:** Univariate analysis of variables associated with endometrial thickness in FET cycles

	**ECT**^**a **^**<8 mm; n=68**	**ECT ≥8 mm; n=206**	**p-value**
***Age (years)***	33.44±4.16	33.93±3.85	0.4
***BMI***^***b ***^***(kg/m***^***2***^***)***	22.7±3.51	25.62±5.54	<0.0001
***History of previous pregnancy (%)***	70.6	87.4	0.001
***History of operative hysteroscopy (%)***	7.4	1.9	0.04
***Duration of estrogen supplementation (days)***	14.9±3	15.1±2.8	0.17
***Fresh cycle ECT (mm)***	10.05±1.89	12.93±3.12	<0.001

a – endometrial cavity thickness; b – body mass index; (χ2 for dichotomous variables, Student’s t-test for continuous variables)

Multivariate analysis was performed to control for the effect of each of the significant characteristics. Similar relationships were found. Lower BMI had a 0.87 odds ratio (OR) of inadequate thickness. Each unit decrease of BMI resulted in an approximate 13% decrease in odds of an adequate endometrium. Prior pregnancy had an OR of 0.4 and operative hysteroscopy had an almost 7-fold increase risk of inadequate thickness. Endometrial thickness in the previous fresh cycle had a 0.58 OR (Table 2).

**Table 2:** Multivariate analysis of factors predictive of inadequate endometrial thickness in FET cycles

	**OR**^**a**^	**95% CI**^**b**^	
		**Lower**	**Upper**
***BMI***^***c***^	0.867	0.794	0.947
***History of previous pregnancy***	0.405	0.179	0.913
***History of operative hysteroscopy***	6.96	1.476	32.824
***Fresh cycle ECT***^***d***^	0.583	0.489	0.695

a – odds ratio; b – confidence interval; c – body mass index; d – endometrial cavity thickness

**Figure 2:**
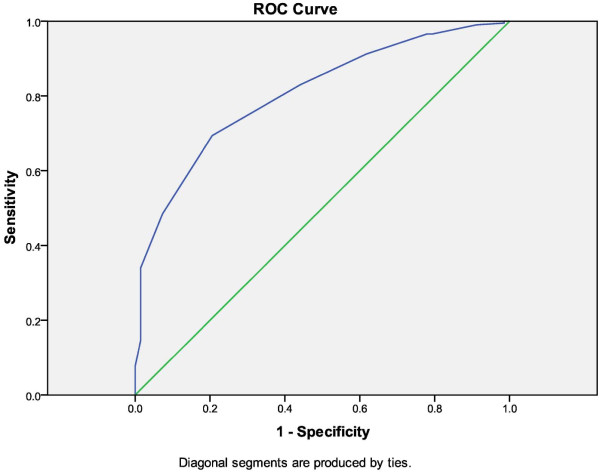
Receiver operator characteristic (ROC) curve for the predictive value of fresh endometrial thickness for FET endometrial thickness.

Because endometrial thickness is potentially modifiable in an FET cycle with variations in estradiol administration, we wanted to determine if a minimum endometrial thickness in fresh IVF cycles would help determine what the endometrial thickness would be after artificial preparation. To do this, we then created an ROC curve that showed that fresh cycle maximum endometrial thickness of 11.5 mm is 80% sensitive and 70% specific for predicting inadequate endometrial thickness in a FET cycle (Figure 2).

## Discussion

Our data indicate that lower BMI, nulligravidity, previous uterine surgery, and a thinner endometrial lining during a fresh cycle are associated with inadequate endometrial thickness. In addition, a fresh cycle maximum endometrial thickness of 11.5 mm was found to be a predictor of inadequate endometrial development in a FET cycle indicating that additional estrogen supplementation may be necessary for these cycles. Although the difference we found in fresh cycle endometrial thickness of 10 mm versus 12.9 mm may not represent a clinically significant difference in implantation rate or pregnancy outcome, it may indicate impaired endometrial development.

Bromer *et al.* also found a positive correlation between endometrial thickness and BMI in patients undergoing clomiphene citrate or FSH treatment [10]. We did not find a significant difference in adequate endometrial development based on age or fertility diagnosis as this group. However, it is possible these patients in our study required additional estrogen during the FET preparation to attain adequate endometrial thickness, and therefore, masking the difference.

When endometrial development is inadequate, it is reasonable to continue estrogen supplementation at the same dose. A potential alternative to unresponsive endometrium is a trial of estradiol via a different route of administration. Because transdermal estradiol does not undergo liver metabolism, there is lower conversion to estrone, and possibly a varied effect on the endometrium [11]. With both interventions, most patients in our study ultimately demonstrated adequate endometrial development and thickness (Figure 1).

Endometrial thickness has long been used as a marker of adequate receptivity of the uterus and as a prognostic factor in embryo transfers. Several studies suggest that pregnancy is less likely when the endometrium is < 8 mm [5-8]. Endometrial thickness < 8 mm does not exclude the possibility of pregnancy, but it is less optimal. In the search for additional markers of success, recent studies have focused on endometrial patterns and sub-endometrial blood flow as a useful adjunct in predicting successful IVF outcomes. A study by Singh *et al.*, looking at endometrial thickness, pattern and sub-endometrial blood flow found a higher pregnancy rate when blood flow to the endometrium was in Zone III (Inner hypoechogenic zone of vascular penetration). They also found that pregnancy rates were highest with an endometrial thickness between 8-10 mm [12]. Chen *et al.*, recently evaluated the combined analysis of endometrial thickness and pattern in predicting outcome of IVF. The author noted that a no triple line endometrial pattern even with a moderate endometrial thickness of 7-14 mm had a detrimental effect on pregnancy outcome, although not the occurrence of pregnancy [13]. They concluded that when a thinner endometrium (< 8 mm) and no triple-line endometrial pattern coexist in an IVF candidate, cryopreservation should be recommended. Despite these findings supporting the importance of endometrial thickness and development, it is important to note that these are surrogate markers of IVF success. Endometrial thickness, in addition to other factors, should be considered.

Several endometrial preparation protocols are utilized, as well as multiple other compounds to attain proper endometrial development. Low-dose aspirin supplementation in women with impaired uterine perfusion may improve uterine blood flow and lead to pregnancy rates similar to women with normal uterine perfusion [14]. Women with uterine fibrosis or thin endometrium for unknown reasons may benefit from prolonged treatment with pentoxifylline and tocopherol [15,16]. Takasaki *et al.* showed improvement in endometrial thickness and uterine radial artery resistance after treatment with either vitamin E, I-arginine or sildenafil citrate [17]. Granulocyte colony-stimulating factor (G-CSF) was also recently shown to increase endometrial thickness [18].

FET is an effective, efficient and affordable means of attaining pregnancy for the patient undergoing IVF. Cryopreservation allows for a decrease in the number of embryos transferred in IVF cycles, while the ability to use preserved embryos in the future leads to an increase in cumulative pregnancy rates. In the patient undergoing FET, predictors of inadequate endometrial development, and potentially inadequate thickness, may help the clinician to be aware and prepared prior to initiation of a new cycle.

Endometrial thickness remains an easily measured and long-employed method of predicting endometrial readiness. In FET cycles in our center, we found that one factor influencing endometrial development is previous endometrial thickness < 11.5 mm. This provides important information for the clinician as these patients may require additional estrogen supplementation. Preparation of the endometrium for FET is the only easily modifiable factor to improve ECT. Other potential factors we identified that can be modified are lower BMI and uterine surgery. Good surgical technique to prevent synechia is critical for adequate endometrial development and should always be practiced. However, in women who have already undergone surgery, this is no longer modifiable. Our data suggests that lower, although normal, BMI is associated with a thinner endometrium. Recommending weight gain would not be appropriate to improve endometrial thickness, and therefore, BMI is not easily modifiable. Our study assists in cycle planning and preparation, and therefore, may decrease prolonged cycles or cancellation and limit costs related to extra visits, ultrasounds and monitoring.

In addition, future study of the employment of ultrasound evaluation of blood flow and pattern would be helpful to determine its utility in making decisions regarding embryo transfer. More research is needed to better understand the mechanism of decreased endometrial thickness as well as how to optimize pregnancy in these patients.

## Conclusions

Endometrial thickness during fresh IVF cycles helps to predict endometrial development in future FET cycles. This may help to identify patients who require additional hormonal support for adequate endometrial development.

## Abbreviations

IVF: In vitro fertilization; FET: Frozen embryo transfer; ECT: Endometrial cavity thickness; BMI: Body mass index; FSH: Follicle stimulating hormone; OR: Odds ratio.

## Competing interests

The authors declare that they have no competing interests.

## Authors’ contributions

PTJ conceived the study, participated in design and data collection, and drafted the manuscript. SBS participated in data collection and manuscript revisions. RRO and VSR participated in critical manuscript revisions. ESJ conceived the study, participated in design, and performed statistical analysis and manuscript revisions. All authors read and approved the final manuscript

## Authors’ information

PTJ is a scholar in the Reproductive Scientist Development Program and a physician in reproductive endocrinology and infertility at the University of Texas Southwestern Medical Center.

SBS is a resident in obstetrics and gynecology at Barnes-Jewish Hospital.

RRO is the division direction of Reproductive Endocrinology and Infertility at Washington University

VSR is the IVF director of the Washington University Reproductive Endocrinology and Infertility clinic

ESJ is a physician in reproductive endocrinology and infertility at Washington University and a Women’s Reproductive Health Research scholar
